# Reassessment of the risk of narcolepsy in children in England 8 years after receipt of the AS03-adjuvanted H1N1 pandemic vaccine: A case-coverage study

**DOI:** 10.1371/journal.pmed.1003225

**Published:** 2020-09-14

**Authors:** Julia Stowe, Nick Andrews, Paul Gringras, Timothy Quinnell, Zenobia Zaiwalla, John Shneerson, Elizabeth Miller

**Affiliations:** 1 Immunisation and Countermeasures, Public Health England, London, England; 2 Statistics and Modelling Economics Department, Public Health England, London, England; 3 Evelina Children’s Hospital, Lambeth, London, England; 4 Respiratory Support and Sleep Centre, Royal Papworth Hospital, Cambridge, England; 5 John Radcliffe Hospital, Headington, England; 6 Royal Papworth Hospital, Cambridge, England; 7 Department of Infectious Disease Epidemiology, Faculty of Epidemiology and Population Health, London School of Hygiene & Tropical Medicine, London, England; Centers for Disease Control and Prevention, UNITED STATES

## Abstract

**Background:**

Early studies of narcolepsy after AS03-adjuvanted pandemic A/H1N12009 vaccine (Pandemrix) could not define the duration of elevated risk post-vaccination nor the risk in children aged under 5 years who may not present until much older.

**Methods/Findings:**

Clinical information and sleep test results, extracted from hospital notes at 3 large pediatric sleep centers in England between September 2017 and June 2018 for narcolepsy cases aged 4–19 years with symptom onset since January 2009, were reviewed by an expert panel to confirm the diagnosis. Vaccination histories were independently obtained from general practitioners (GPs). The odds of vaccination in narcolepsy cases compared with the age-matched English population was calculated after adjustment for clinical conditions that were indications for vaccination.

GP questionnaires were returned for 242 of the 244 children with confirmed narcolepsy. Of these 5 were under 5 years, 118 were 5–11 years, and 119 were 12–19 years old at diagnosis; 39 were vaccinated with Pandemrix before onset. The odds ratio (OR) for onset at any time after vaccination was 1.94 (95% confidence interval [CI] 1.30–2.89), The elevated risk period was restricted to onsets within 12 months of vaccination (OR 6.65 [3.44–12.85]) and was highest within the first 6 months. After one year, ORs were not significantly different from 1 up to 8 years after vaccination. The ORs were similar in under five-year-olds and older ages. The estimated attributable risk was 1 in 34,500 doses. Our study is limited by including cases from only 3 sleep centers, who may differ from cases diagnosed in nonparticipating centers, and by imprecision in defining the centers’ catchment population. The potential for biased recall of onset shortly after vaccination in cases aware of the association cannot be excluded.

**Conclusions:**

In this study, we found that vaccine-attributable cases have onset of narcolepsy within 12 months of Pandemrix vaccination. The attributable risk is higher than previously estimated in England because of identification of vaccine-attributable cases with late diagnoses. Absence of a compensatory drop in risk 1–8 years after vaccination suggests that Pandemrix does not trigger onsets in those in whom narcolepsy would have occurred later.

## Abstract 2

Julia Stowe and co-workers study narcolepsy among children in England who received a pandemic influenza vaccine

## Author summary

**Why was this study done?:**

- The AS03-adjuvanted H1N1 pandemic vaccine Pandemrix was shown to be associated with an excess of cases of narcolepsy, but studies could not determine for how long after vaccination the risk was elevated nor whether there was a later compensatory reduction.
- These early studies were unable to assess the risk in children vaccinated under 5 years of age because of the long diagnostic delays in this age group.
- There were also concerns that the elevated risk shown in these early studies may have resulted from ascertainment bias due to the earlier presentation and diagnosis in cases known to be vaccinated. Addressing these questions requires studies that have a long follow-up interval after vaccination.

**What did the researchers do and find?:**

- Records at 3 large pediatric sleep centers in England were searched to find cases of narcolepsy aged 4–19 years with symptom onset since January 2009. Independently Pandemrix vaccination histories of these cases were obtained. The proportion of cases vaccinated before onset were compared with that in the age-matched population at the time of symptom onset in each case.
- A 7-fold elevated risk of onset of narcolepsy after Pandemrix was found, which was confined to the first year after vaccination, with the highest risk within 6 months. No evidence of a compensatory drop in risk to below one between 1 and 8 years after vaccination was seen.
- Vaccine-associated risk was similar in children vaccinated under 5 years or at an older age.

**What do these findings mean?:**

- The long follow-up interval makes it unlikely that bias because of earlier diagnosis in vaccinated cases generated the elevated risk reported after Pandemrix and provides strong confirmatory evidence of a causal association.
- The new information on the period of elevated risk after Pandemrix will help clinicians decide on causality in individual narcolepsy cases with a history of vaccination.
- The lack of a drop in risk below baseline after a year indicates that Pandemrix was unlikely to have accelerated the onset in those who were destined to develop narcolepsy later.

## Introduction

In August 2010, concerns were raised in Finland and Sweden about a possible association between narcolepsy and the AS03-adjuvanted H1N1 pandemic vaccine Pandemrix following reports of an excess of cases in recently vaccinated children [1,2]. A subsequent cohort study in Finland reported a 13-fold increased risk of narcolepsy following Pandemrix in children aged 4 to 19 years, almost of whom had onset within 6 months [3].

Pandemrix was the predominant H1N1 vaccine used in Europe, with over 30 million individuals vaccinated. In the UK, Pandemrix was used from October 2009 for individuals considered at high risk followed by children under 5 years of age from December onwards. It was used again in the UK in the 2010/11 season because of a shortage of seasonal influenza vaccine. Over 5 million UK subjects received Pandemrix [4].

To investigate the association reported from Finland, the Health Protection Agency (now Public Health England) performed a study in 2011 in sleep centers in England. This identified a 14-fold increased risk in those vaccinated with Pandemrix aged under 19 years with an estimated attributable risk of 1.9 per 100,000 doses [5]. Subsequently, Public Health England investigated the association in adults and found a 9-fold increased risk with an attributable risk of 0.6 per 100,000 doses [6]. Increased risks in children and adults have also been reported in other European countries where Pandemrix was used, but no association has been seen with other pandemic or seasonal vaccines [7]. Despite the consistency of the findings, it has been suggested that the elevated risk estimates reported in these studies are biased due to more rapid diagnosis in cases known to have been vaccinated [8,9].

Narcolepsy cases usually have a long period between symptom onset and diagnosis. Because the studies reported to date had relatively short follow-up periods, usually less than 2 years, cases with an extended period between symptom onset and diagnosis or with a long onset interval after vaccination would not have been identified. In addition, narcolepsy is rarely diagnosed under the age of 5 years, so any risk in children vaccinated at an early age would not have been assessed. We therefore conducted a further pediatric study in England 8 years after Pandemrix was first used to re-evaluate the risk when including cases with a delayed diagnosis, to investigate the postvaccination period in which the risk is elevated and to assess the risk in those too young to be diagnosed at the time of the previous study. In addition, long-term risk estimates were used to assess whether the vaccine may have triggered early onset in cases that would otherwise have occurred later.

## Methods

The study protocol and preplanned analyses are provided in S1 Text. The intention was to include 5 study sites, but as sufficient cases had been recruited to meet the sample size requirements at the first 3 centers, the remaining 2 sites were not included. The STROBE check list for observational studies is provided in S1 STROBE checklist.

### Case ascertainment and validation

Hospital episode statistics (HES) in England [10] with an ICD10 code G47.4 for narcolepsy were used to identify the 3 major pediatric centers for narcolepsy diagnoses in England. The 3 centers, all which participated in the 2011 study, were visited between September 2017 and June 2018. The age distribution and the sex ratio of HES cases at the 3 centers were similar to that at other centers not visited (S1 Fig).

**Fig 1:**
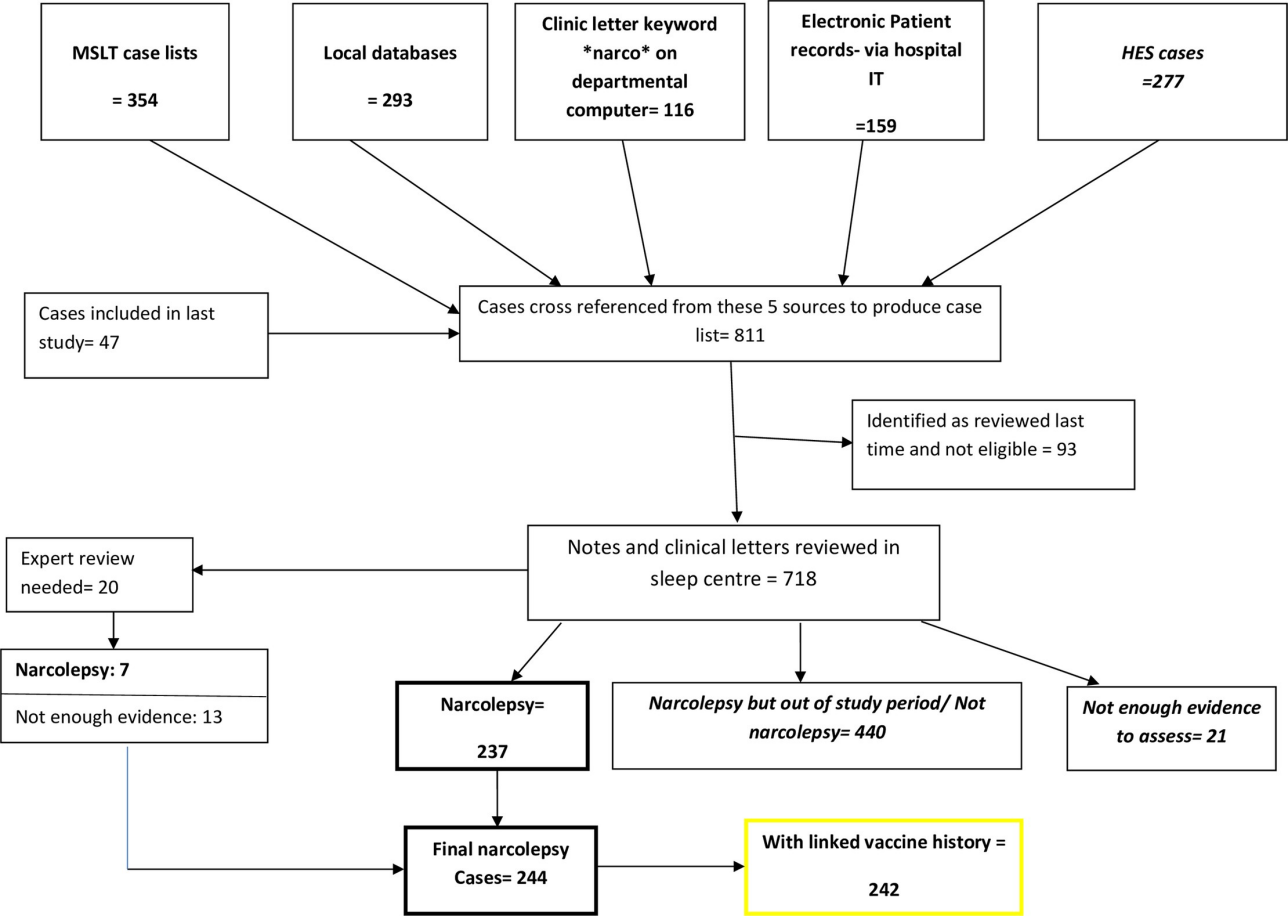
Flow diagram for identification of study participants. HES, Hospital episode statistics; IT, information technology; MSLT, multiple sleep latency test.

Cases aged 19 years or under at the time of diagnosis were identified using local databases, multiple sleep latency test (MSLT) case lists, electronic patient records, and a clinic letters search for the keyword *narco*. The case lists from the local search, HES, and the previous study were merged and de-duplicated using NHS number or surname and date of birth. These potential cases were then reviewed using electronic medical records to establish symptom onset date, clinical history, and sleep study results with any missing information supplemented by review of paper case notes (Fig 1).

The anonymized clinical information from each center was then evaluated by a review panel of sleep medicine clinicians (authors PG, TQ, JShn, ZZ) prior to ascertainment of vaccination status. Cases with a clear history of excessive daytime sleepiness (EDS) and cataplexy with a positive MSLT or low levels of hypocretin in cerebrospinal fluid (CSF) were not sent to the panel for review. The panel used the International Classification of Sleep Disorders Third Edition (ICSD-3) [11] and categorized each case as Type 1 (cataplexy and/or low hypocretin levels), Type 2 (no cataplexy, hypocretin normal or not measured), possible, or exclude. A consensus of 3 of the 4 panel members was required to assign a final diagnosis.

A questionnaire was sent to general practitioners (GPs) for patients categorized as Type1 or Type 2 narcolepsy requesting information on EDS or cataplexy symptoms, date these symptoms first occurred, and date of first consultation about these symptoms. Pandemic influenza vaccine dates were also requested together with batch/lot number and whether the patient was in an influenza vaccine eligibility risk group in 2009.

### Index dates

The date of first symptoms, date of first healthcare contact, and date of diagnosis were determined from the medical records at the sleep center or from the GP questionnaire. The date of first symptom was defined as the earliest date of EDS, or cataplexy if that was the presenting symptom, as reported by the GP or mentioned in the hospital notes. Date of diagnosis was the date of the sleep test or if not done the date of the first letter from the sleep center confirming a narcolepsy diagnosis. A best first symptom date and earliest and latest possible first symptom date were assigned using all the information on the timing of the onset before the vaccine status was sought. The best estimated date was used for the main analysis and the earliest and latest dates as a sensitivity analysis. In general, the month midpoint (15th) was used unless a very specific date was given.

### Statistical analysis

The association between Pandemrix and narcolepsy was assessed using the case-coverage method [5,6,12], which compares the proportion of cases vaccinated with the proportion vaccinated in the population from which the cases arose (i.e., vaccine coverage in that population). With this method, the population for which coverage is obtained will contain both cases and noncases, but for rare conditions such as narcolepsy, this will approximate to the coverage in noncases. Where coverage is rapidly changing over time and varies by age and clinical risk group, as with the roll out of Pandemrix, the coverage population needs to be matched by these variables. To obtain for each case the age-matched population coverage in England according to risk group status by the time of the index date in the case (i.e., symptom onset or first healthcare contact), individual anonymized vaccination records were obtained from the Clinical Practice Research Datalink (CPRD) [13]. This contains patient-level information for over 1,500 GP practices in the UK, including dates of Pandemrix doses given to children aged 6 months to 19 years for both the 2009/10 and 2010/11 seasons as well as data on clinical risk group. CPRD data for England were used to derive cumulative Pandemrix vaccine coverage figures stratified by calendar day, age (6–11 months and then individual yearly age groups to 19 years as at January 2010) and whether in a clinical risk group targeted to receive the influenza vaccine (S2 Fig). Narcolepsy cases were similarly allocated to a risk group based on the information provided by the GP on clinical conditions considered high risk for influenza as of 2009.

The association was calculated as the odds ratio (OR) for vaccination in the cases compared with the matched population. This was done with logistic regression with the outcome as vaccinated (yes/no) in the cases and with an offset for the log odds of the matched coverage. As the outcome is rare, ORs approximate to relative risks. The primary analysis used the first symptom date as the index date with the symptom onset date from October 2009 to the date diagnosed by the sleep center visit date. This primary analysis was based on ever receiving Pandemrix_._ and was stratified by age group into <5, 5–19 years and by onset interval (<12 months, 12–23 months, 2–3 years, and 4–8 years after vaccination). All analyses were conducted as specified in the preplanned analysis (S1 Text) with an additional post-hoc analysis in which the risk in the first 2 years post-vaccination was further split into within 12 months and 12–23 months, and a figure splitting the first 12 months into 0–5 and 6–11 months was generated. Sensitivity analyses were carried out adjusting the vaccine coverage estimates by a relative ±20%; no risk group adjustment; using earliest and latest symptom onset dates; excluding unvaccinated cases in which the GP may have been unable to verify their status; and using first healthcare contact date for which risk was assessed within 24 months, 2–3 years, or 4–8 years after vaccination.

A statistically significant elevated or reduced risk (*p* < 0.05) was inferred if the 95% confidence intervals (CIs) for the OR did not contain 1. Analyses were conducted in Stata (StataCorp. 2015. Stata Statistical Software: Release 14. College Station, TX: StataCorp LP: https://www.stata.com/).

### Ethics approval

Public Health England is able to process identifiable data under Regulation 3 of The Health Service (Control of Patient Information) (Secretary of State for Health, 2002). This is for purposes related to communicable diseases and other risks to public health and includes the delivery, efficacy, and safety of immunization programs and adverse reactions to vaccines and medicines.

## Results

### Study cases

Case notes for 811 potential narcolepsy patients were identified at the 3 centers. This included 140 patients reviewed as part of the previous study of which 93 were not eligible for this study as they had onset before 2009. The case notes for the remaining 718 patients were reviewed (Fig 1); of these, 440 had their first symptoms of narcolepsy before January 2009 or did not have narcolepsy, and 21 did not have enough information available to assess the diagnosis. These 461 patients were excluded. Of the remaining 257 cases, 237 were considered definite narcolepsy, and for 20, anonymized case notes were sent to the expert panel for review. The panel could confirm a diagnosis of narcolepsy for 7 of these cases, giving a total of 244 of which 204 were categorized as type 1 narcolepsy and 40 as type 2 narcolepsy.

### Vaccination history

The GP questionnaire was returned for 242 of the 244 patients; the remaining 2 patients were excluded from further analyses. Pandemrix vaccine and date were reported for 44 patients with a confirmatory batch number provided for 37. Of the 44 Pandemrix recipients, 39 were vaccinated before and 5 after symptom onset. Two patients had received 2 doses, and for these, the date of the last dose was used in the analysis as symptom onset occurred after the second dose. There were 17 individuals reported as unvaccinated where the GP indicated that they were not registered in their practice in 2009 and that record transfer did not occur, and a further 23 reported as unvaccinated for whom date of registration at the practice was not provided. These 40 cases were retained for the primary analysis and counted as unvaccinated on the assumption that an individual who did receive the vaccine and then developed narcolepsy would have ensured this information was transferred when registering with a new GP. Of the 26 patients reported to be in a risk group eligible for Pandemrix in 2009, 13 received the vaccine.

**Fig 2:**
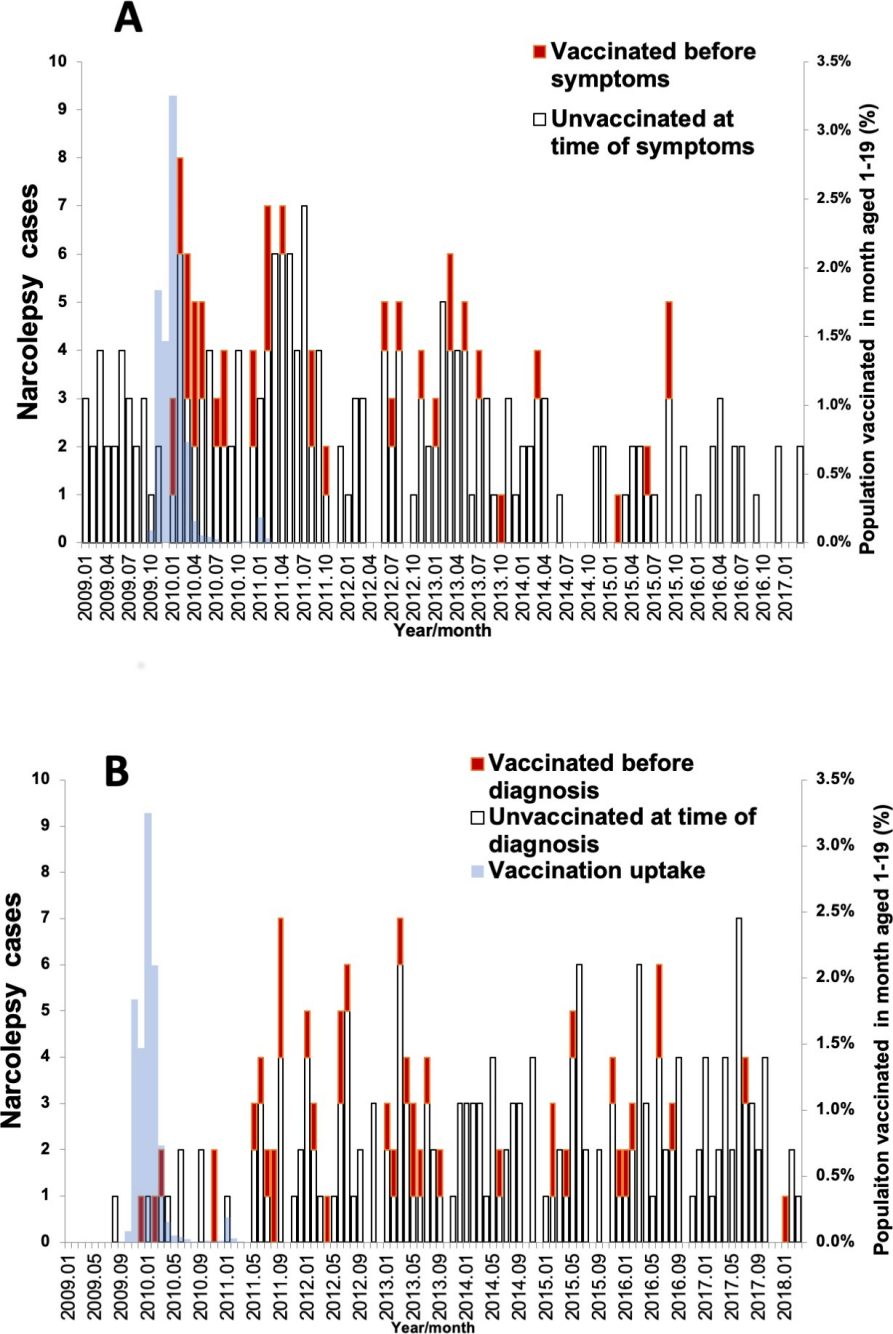
Timing of onset (A) or diagnosis (B) for the 242 narcolepsy cases by vaccination status and monthly vaccine uptake in the age-matched population.

The demographic and clinical details of the 242 cases are shown in Table 1 by vaccination status. Only 5 (2%) were under 5 years of age at the time of diagnosis. All cases with Type 1 narcolepsy also had cataplexy. Of the 39 cases with onset after vaccination, 36 (92%) had type 1 narcolepsy compared with 168 (82%) of those unvaccinated or vaccinated after onset. Of the 111 (46%) tested for human leucocyte antigen (HLA) DQB1 *06:02, all were positive. Fig 2A shows the symptom onset date in the 242 cases, indicating the 39 who received Pandemrix before onset. The decline in the number of narcolepsy cases with time in Fig 2A is consistent with the lag time between onset and diagnosis; when plotted by diagnosis date (Fig 2B), the cases show an even distribution over time.

### Assessment of time to diagnosis by vaccination status

**Table 1:** Summary of demographic and clinical features of the 242 cases by vaccination status.

Feature	Level	Unvaccinated (*n* = 198)	Vaccinated before onset (*n* = 39)	Vaccinated after onset (*n* = 5)	Total (*n* = 242)
Age at September 2009 (years)	<5	53	24	2	79
	5–11	109	12	2	123
	12–19	36	3	1	40
Age at onset (years)	<5	13	7	2	22
	5–11	115	26	1	142
	12–19	70	6	2	78
Age at diagnosis (years)	4	4	1	0	5
	5–11	86	29	3	118
	12–19	108	9	2	119
Sleep Center (date visited)	Oxford (June 2018)	53	15	0	68
	Papworth (Jan 2018)	25	2	1	28
	St Thomas' (Sep 2017)	120	22	4	146
Gender	Male	103	24	1	128
	Female	95	15	4	114
Risk group for influenza vaccine in 2009	Yes	13	10	3	26
	No	138	21	2	161
	Not known	47	8	0	55
Diagnostic Category	Type I	163	36	4	203
	Type II	35	3	1	39
HLA DQB1*06:02	Positive	88	23	0	111
	Not reported	110	16	5	131
CSF measurement	Indicative of narcolepsy (≤110 pg/ml)	11	10	1	22
	Not reported	187	29	4	220

CSF, cerebrospinal fluid; HLA, human leucocyte antigen.

**Fig 3:**
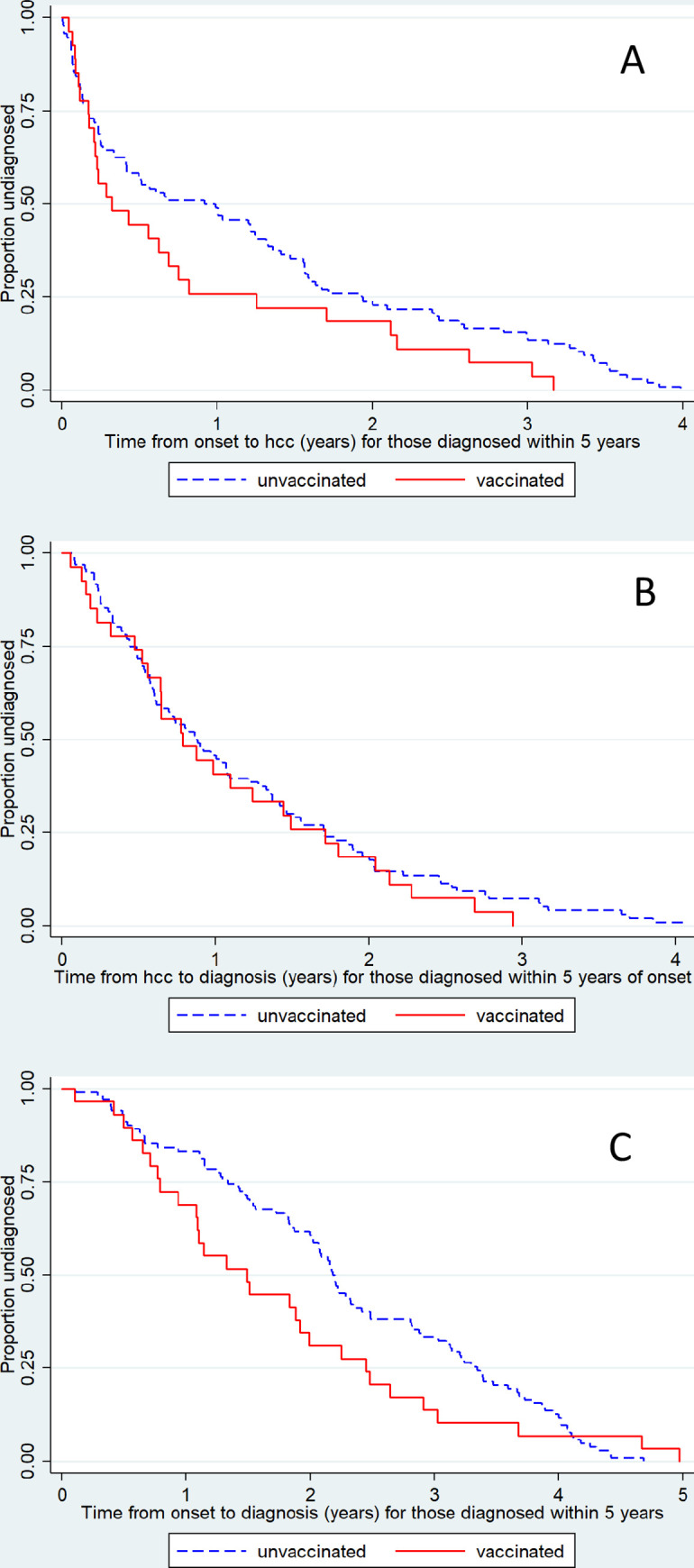
Interval from symptom onset to first hcc (A), from first hcc to diagnosis (B) and from symptom onset to diagnosis (C). In cases with onset from January 2009 to September 2012 and with at least 5 years of follow-up time. hcc, healthcare contact.

A total of 29 vaccinated and 101 unvaccinated cases with onset from January 2009 to September 2012 with at least 5 years of time in the study were included in this assessment. There was some evidence of a shorter interval from the onset of symptoms to the patients’ first healthcare contact in the vaccinated cases (Fig 3A) although no difference in the interval from their first healthcare contact to their diagnosis (Fig 3B). As a result, there was a shorter overall interval from onset to diagnosis in vaccinated cases (Fig 3C), the median interval being 1.49 years for a vaccinated case compared with 2.19 years for an unvaccinated case (*p* = 0.024).

### Case-coverage analysis

Table 2 shows the results using date of symptom onset stratified by age group and interval to symptom onset. There were 200 cases eligible for this analysis after excluding 3 cases for whom only a latest onset date could be allocated, 26 whose onset was before the vaccine was available in mid-October 2009 and 13 who were aged <6 months on January 1, 2010, and were not eligible for vaccine that year.

**Table 2:** Association between narcolepsy and Pandemrix vaccination by timing of symptom onset relative to vaccination and age in 2009.

Age at September 2009	Interval before onset	Vaccinated patients	Total patients eligible for vaccination in interval before index date	Average matched coverage^a^	Odds ratio (95% CI)
<5 years	Any time	24	63	0.266	1.77 (1.04–3.00)
	<12 months	8	23	0.122	6.76 (2.10–21.72)
	12–23 months	3	28	0.098	1.13 (0.30–4.33)
	2–3 years	9	30	0.227	1.53 (0.66–3.54)
	4–8 years	4	17	0.256	0.88 (0.28–2.83)
5–19 years	Any time	15	137	0.060	2.18 (1.20–3.97)
	<12 months	10	83	0.033	6.60 (2.97–14.68)
	12–23 months	3	62	0.041	1.24 (0.35–4.42)
	2–3 years	1	49	0.049	0.37 (0.05–2.85)
	4–8 years	1	23	0.028	1.58 (0.21–11.83)
All ages	Any time	39	200	0.125	1.94 (1.30–2.89)
	<12 months	18	106	0.052	6.65 (3.44–12.85)
	12–23 months	6	90	0.058	1.19 (0.47–2.99)
	2–3 years	10	79	0.117	1.12 (0.54–2.34)
	4–8 years	5	40	0.125	1.00 (0.36–2.80)

^a^Coverage in population by index date in case matched for risk group status and age at September 2009.

CI, confidence interval.

**Fig 4:**
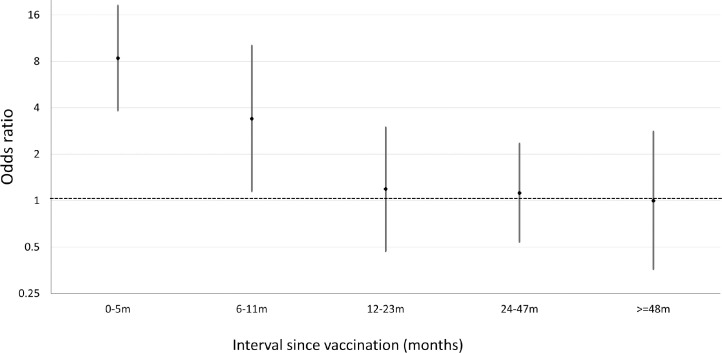
Odds ratio of vaccination in narcolepsy cases compared with age-matched population prior to symptom onset according to time interval of onset since vaccination.

The OR of vaccination at any time before onset in those with narcolepsy compared with the matched population by the same index date, was significantly elevated both in those who received the vaccine before 5 years of age and those vaccinated when aged 5–19 years, with similar ORs for the 2 age groups. In both, the period of elevated risk was confined to onsets within 12 months of vaccination. Within the first 12 months the risk was highest in those with onset within 6 months of vaccination (Fig 4).

There was no evidence of a reduced risk in any time period after 12 months. In the sensitivity analyses (Table 3) not matching on risk group gave similar results with an OR of 7.15 (95% CI 3.89–13.14) for onsets within 12 months compared with 6.65 (95% CI 3.44–12.85) with risk group matching (Table 2). Exclusion of the 17 patients reported to be unvaccinated but without transferred GP records and the 23 whose registration status in 2009 was unknown increased ORs as expected. Using the earliest possible symptom onset date reduced the effect (OR for onset within 12 months 3.30 [95% CI 1.61–6.74]) and using the latest date increased the effect (OR 8.33 [95% CI 4.36–15.91]) as some patients changed vaccination status. The results were broadly similar to those in the main analysis when population vaccine coverage was varied by a relative ±20% (e.g., 25% coverage observed in CPRD increased to 30% or reduced to 20%). Analyses using date of first healthcare contact showed an elevated risk within 2 years, consistent with the delay between onset and initial consultation.

**Table 3:** Sensitivity analyses for the association between narcolepsy and Pandemrix vaccination by timing of index date relative to vaccination.

Analysis	Interval before index date	Vaccinated patients	Total patients eligible for vaccination in interval before index date	Average matched coverage	Odds ratio (95% CI)
Not matching on risk group	Any time	39	200	0.114	2.12 (1.44–3.13)
	<12 months	18	106	0.043	7.15 (3.89–13.14)
	12–23 months	6	90	0.051	1.35 (0.55–3.34)
	2–3 years	10	79	0.105	1.29 (0.62–2.66)
	4–8 years	5	40	0.131	0.94 (0.35–2.58)
Dropping those without data transfer at GP or not known if registered	Any time	39	172	0.124	2.54 (1.67–3.86)
	<12 months	18	93	0.051	8.74 (4.48–17.05)
	12–23 months	6	77	0.058	1.47 (0.57–3.82)
	2–3 years	10	66	0.115	1.48 (0.69–3.19)
	4–8 years	5	33	0.135	1.18 (0.41–3.40)
Using earliest onset date as index date	Any time	33	188	0.124	1.65 (1.08–2.53)
	<12 months	13	105	0.055	3.30 (1.61–6.74)
	12–23 months	6	91	0.045	1.63 (0.64–4.16)
	2–3 years	10	74	0.124	1.13 (0.54–2.36)
	4–8 years	4	31	0.138	0.91 (0.29–2.88)
Using latest onset date as index date	Any time	41	215	0.122	1.94 (1.31–2.87)
	<12 months	19	109	0.049	8.33 (4.36–15.91)
	12–23 months	5	95	0.058	0.89 (0.33–2.38)
	2–3 years	12	95	0.098	1.43 (0.72–2.85)
	4–8 years	5	48	0.127	0.76 (0.28–2.07)
Decreasing uptake by a relative 20%	Any time	39	200	0.100	2.57 (1.73–3.83)
	<12 months	18	106	0.042	8.57 (4.46–16.45)
	12–23 months	6	90	0.047	1.57 (0.62–3.93)
	2–3 years	10	79	0.093	1.50 (0.72–3.13)
	4–8 years	5	40	0.100	1.34 (0.48–3.75)
Increasing uptake by a relative 20%	Any time	39	200	0.150	1.51 (1.00–2.25)
	<12 months	18	106	0.062	5.37 (2.76–10.44)
	12–23 months	6	90	0.070	0.93 (0.37–2.36)
	2–3 years	10	79	0.140	0.86 (0.41–1.81)
	4–8 years	5	40	0.150	0.77 (0.27–2.16)
Using first healthcare contact as the index date	Any time	42	217	0.122	2.00 (1.36–2.96)
	<24 months	21	99	0.071	5.83 (3.12–10.71)
	2–3 years	11	107	0.083	1.35 (0.66–2.77)
	4–8 years	10	91	0.114	0.95 (0.46–1.94)

CI, confidence interval; GP, general practitioner.

### Attributable risk

The calculation for the vaccine-attributable risk is based on the OR for cases with onset within 12 months of vaccination and with adjustment for risk group status (6.65 as shown in Table 2). Using the OR to approximate relative risk (RR), the attributable fraction ([RR−1]/RR) is 5.65/6.65 (85.0%), which, from the total of 18 cases with onset within 12 months (Table 2), gives 15.3 vaccine-attributable cases.

To estimate the size of the catchment population of the 3 study centers, we identified the number of children in England aged ≤19 years with a diagnosis of narcolepsy (ICD10 code G47.4) in HES for the period January 1, 2008, to March 31, 2017 (*n* = 646). The number from HES in the 3 centers for the same period was 263. Thus, we estimated that the 3 study centers receive referrals from 263/646 (41%) of the pediatric population in England. The number of doses received by the population covered by these 3 sleep centers was estimated from the CPRD uptake in each age group × 0.41 × population in each age group in England (Office for National Statistics population [14]), which gave a total of 528,001 doses (S1 Data). The attributable risk is therefore 15.3/528,001 = 2.9 per 100,000 doses or 1 per 34,500 doses.

## Discussion

This study conducted 8 years after Pandemrix was first used in England allowed the risk of narcolepsy to be re-evaluated by the inclusion of cases in whom the diagnosis occurred after July 2011, the date at which follow-up was censored in the earlier study [5]. It confirmed an elevated risk after Pandemrix, which was confined to those with onset within the first 12 months with a return to baseline thereafter. We found no evidence of a compensatory drop in risk to below 1 after 12 months as might be expected if the vaccine had triggered onsets in cases destined to occur later. Our study also showed that children who received the vaccine under 5 years of age were at a similar increased risk to those vaccinated when older.

The 8-fold increased risk as measured by the OR for cases with onset within 6 months after vaccination (Fig 4) was lower than that in our earlier study in which a 16-fold increased risk was found for the same onset interval [5]. Because all the eligible cases at the 3 centers from the earlier study were included in the current analysis, this suggests some bias toward preferential ascertainment of vaccinated cases in our earlier study. Although the interval between first healthcare contact and diagnosis was similar in vaccinated and unvaccinated cases, the former had a shorter interval from onset to first healthcare contact (Fig 3), which, with the short follow-up interval in the earlier study, would have biased upwards the risk estimates. This earlier contact with healthcare may reflect more severe or abrupt onset in vaccinated cases or knowledge of the putative association prompting earlier consultation, even though the first evidence of heightened public interest in narcolepsy (as judged by Google searches in the UK) did not occur until December 2011 [15]. Despite the lower ORs in the current study, the estimated attributable risk (1 per 34,500 doses) was higher than estimated in the previous study (around 1 in 55,00 doses) because of the inclusion of a substantial number of vaccine-attributable cases with a diagnosis after July 2011.

The strengths of our study lie in the exhaustive search for potential narcolepsy cases at the participating study centers using multiple data sources; the long follow-up interval, allowing the inclusion of cases with delayed diagnoses; the independent verification of vaccination histories from GP records; and the use of internationally agreed diagnostic criteria with case inclusion decided on case note review without knowledge of vaccination status. Furthermore, our study was adequately powered to look at the risk in sequential time periods post-vaccination. The conclusions from the main analysis (Table 2) were essentially the same in the sensitivity analysis (Table 3). Our study should therefore help dispel the concerns raised about the extent to which ascertainment bias, recall bias, and bias by indication affected the results in the earlier studies in Finland, Sweden, and the UK [8,9].

Our study has a number of limitations. Our case ascertainment was restricted to 3 large pediatric centers in southern England, and although the cases at these centers had similar demographic features to cases attending other sleep centers in England (S1 Fig), the patient populations may have differed in other ways we were unable to measure. Also we were unable to geographically define the catchment population of these 3 centers when estimating absolute risks so had to assume that the proportion of narcolepsy cases in England seen at these centers reflected the proportion of the pediatric population in England in the catchment area of the centers. There is the possibility that some onset dates reported in the clinical notes may have been influenced by patients’ knowledge of their vaccination status, particularly those that presented after the association with Pandemrix started to attract public attention in December 2011 [5]. However, using the more objective date of first healthcare contact as supplied by GPs, an excess risk was still apparent within 2 years and at any time after vaccination. Bias due to earlier presentation of vaccinated cases up to 8 years after vaccination seems unlikely. Our conclusion that Pandemrix did not accelerate onsets in cases that would otherwise have occurred later was based on the ORs after 12 months being consistently close to one. However, there were relatively few cases in these later periods, and a small reduction or elevation in risk could not be excluded. Although a Swedish study found a small elevated risk of onset in the second year after vaccination, this was based on only 19 cases and was conducted within 3 years of Pandemrix use so could not include those with late diagnoses [16].

The epidemiological evidence for a causal association between Pandemrix and development of narcolepsy in children based on our findings, and those of earlier studies in European is compelling [7,17]. However, the mechanism behind the association and whether it is exclusive to Pandemrix remains unclear. A multicountry study of adjuvanted H1N1 pandemic vaccines failed to find an increased risk in children after Arepanrix, a Canadian-manufactured AS03 vaccine similar to Pandemrix, or Focetria, an MF59-adjuvanted vaccine [18]. However, these estimates were based on single studies in Ontario and Taiwan, respectively. An earlier study of Arepanrix in Quebec reported a relative risk of 4.32 (95% CI 1.50–11.2) in a cohort analysis in under 20-year-olds, though with a very low attributable risk of around 1 per million doses [19]. Further studies are needed to confirm whether the association between narcolepsy and pandemic H1N1 vaccines is confined to Pandemrix.

Various hypotheses about the potential pathogenic mechanism underlying the association between Pandemrix and narcolepsy have been advanced with the evidence suggestive of an autoimmune mechanism. [20,21]. It has also been suggested that H1N1 pandemic influenza infection itself can cause narcolepsy based on an increase in reported cases in Beijing and Taiwan in the wake of the 2009 pandemic [22,23]. However, this has not been seen in other settings. The recently advanced hypothesis that prior H1N1 infection is a necessary condition for Pandemrix-induced narcolepsy (the so-called 2-hit hypothesis) is, at present, purely speculative [24].

Adjuvanted vaccines were shown to have superior protective efficacy to unajuvanted vaccines in the 2009 pandemic [25]. The case for their use in a future pandemic is therefore strong, particularly if caused by a strain with a high case-fatality rate or to which the population is immunologically naive. An AS03-adjuvanted H5N1 vaccine has been licensed by the Food and Drug Administration in the United States for use in their national stockpile [26], although its licensure is restricted to adults for whom the risk of narcolepsy after Pandemrix is lower than in children [7]. However, without a proper understanding of the mechanism whereby Pandemrix can cause narcolepsy and the role, if any, of wild virus influenza infection, it is currently difficult to assess whether there might be a risk from future pandemic vaccines containing AS03 but with a different pandemic strain.

## Supporting information

S1 STROBE checklist STROBE, Strengthening the Reporting of Observational Studies in Epidemiology. (DOCX)

S1 Text Protocol and preplanned analysis. (DOC)

S1 Fig Age and sex distribution of cases with an HES diagnosis of narcolepsy (ICD10 G47.4) at the 3 study centers and other sleep centers in England September 2017 to June 2018. HES, hospital episode statistics; ICSD-3, International Classification of Sleep Disorders Third Edition.
(TIF)

S2 Fig Calculation of coverage in matched population. (DOCX)

S1 Data Estimation of doses given to the population covered by the 3 study centers. (DOCX)

## Abbreviations

CI: confidence interval
CPRD: Clinical Practice Research Datalink
GP: general practitioner
HES: hospital episode statistics
HLA: human leucocyte antigen
OR: odds ratio
RR: relative risk
