# Supplementary material for: Reassessment of the risk of narcolepsy in children in England 8 years after receipt of the AS03-adjuvanted H1N1 pandemic vaccine: A case-coverage study
Source: PLoS Med. 2020 Sep 14;17(9):e1003225. doi: 10.1371/journal.pmed.1003225 (PMC7489954; doi:10.1371/journal.pmed.1003225)

**Enhanced Surveillance of Narcolepsy in children and the possible association with Pandemrix in England**

**Protocol and Statistical Analysis Plan for a multi-centre study in England**

**March 2019 (updated pre-analysis version)**

**E.Miller, J.Stowe N.Andrews**, Public Health England, London, UK

**1. Introduction**

Narcolepsy is a disabling and chronic sleep disorder which presents itself with excessive daytime sleepiness of sudden onset in conjunction with an inability to remain asleep for long periods during the night. A specific symptom of narcolepsy is cataplexy which is a transiently bilateral loss of muscle tone whilst remaining conscious. This is initiated by a change in emotions such as laughing. The prevalence of narcolepsy with cataplexy has been shown to between 25 and 50 per 100,000 people with one study giving an incidence of 0.74 per 100,000 person years (1). Age of onset usually occurs between 15 and 40 years and symptoms develop gradually so time from onset to diagnosis can be many years.

In August 2010 concerns were raised in Finland and Sweden about a possible association between narcolepsy and Pandemrix. A subsequent cohort study in Finland reported a 13 fold increased risk of narcolepsy following Pandemrix in children aged 4 to 19 years, the majority of whom had onset within 3 months of vaccination and almost all within 6 months (2-4).

Pandemrix was used in the UK from October 2009 where people within a seasonal flu vaccine risk group and pregnant women were offered the vaccine followed by children under 5 years of age from December onwards (figure 1). Approximately 6 million subjects in the UK have been vaccinated with the H1N1 vaccine Pandemrix. It was the predominant H1N1 vaccine used within the EU.

To assess the risk identified in Finland the Health Protection Agency (now Public Health England) performed a study in sleep centres in England where the majority of children are seen. This study identified a 14 fold increased risk in those vaccinated with Pandemrix (5) with the attributable risk estimated to be 1.9 per 100 000 doses. Subsequent to this Public Health England used similar methodology to look at the risk in adults where a 9 fold risk was reported with an attributable risk of 0.6 per 100,000 doses (6). Increased risks have also been reported in Sweden France, Ireland and Norway (7-12).

Narcolepsy is known to have a long period between symptoms and diagnosis (typically many years). The HPA childhood narcolepsy study identified cases diagnosed up to about the end of 2011 which means many cases with onset in 2009, 2010 and 2011 will not have been diagnosed as well as further cases with onset at greater intervals since vaccination. In addition cases are rarely diagnosed aged under about 4 due to the age of the child. There are a number of possible consequences of this:

1. The attributable risk may be under estimated.
2. The post vaccination interval of increased risk is unclear as follow up was a maximum of 2 years. The attributable risk reported in our study currently relates to any time after vaccination. Using a short interval such as 6 months was found not to be adequate in our study.
3. Accelerated diagnosis in vaccinated compared to unvaccinated children could lead to over estimation of the risk. To minimise this in the HPA study an analysis was done with only cases diagnosed prior to media attention (prior to July 2011).
4. Any risk in children aged <5 when vaccinated may not have been identified. This group was universally targeted in the UK so form the majority of the vaccinated group in children.

Updating the childhood study in 2017/2018, over eight years since Pandemrix vaccine use will allow us to address the issues listed above. Updating the relative and attributable risk for narcolepsy at any time after vaccination will address points i) and iii), as will comparing time to diagnosis in vaccinated and unvaccinated cases. An analysis of the relative risk (measured as an odds ratio) by time since vaccination will also address points ii) and iii) since a more delayed diagnosis in unvaccinated will lead to odds ratios below one at longer intervals since vaccination. The analysis will also address point iv), particularly if stratified by age at vaccination.

Figure 1: Pandemrix vaccine uptake in England by age and risk group.


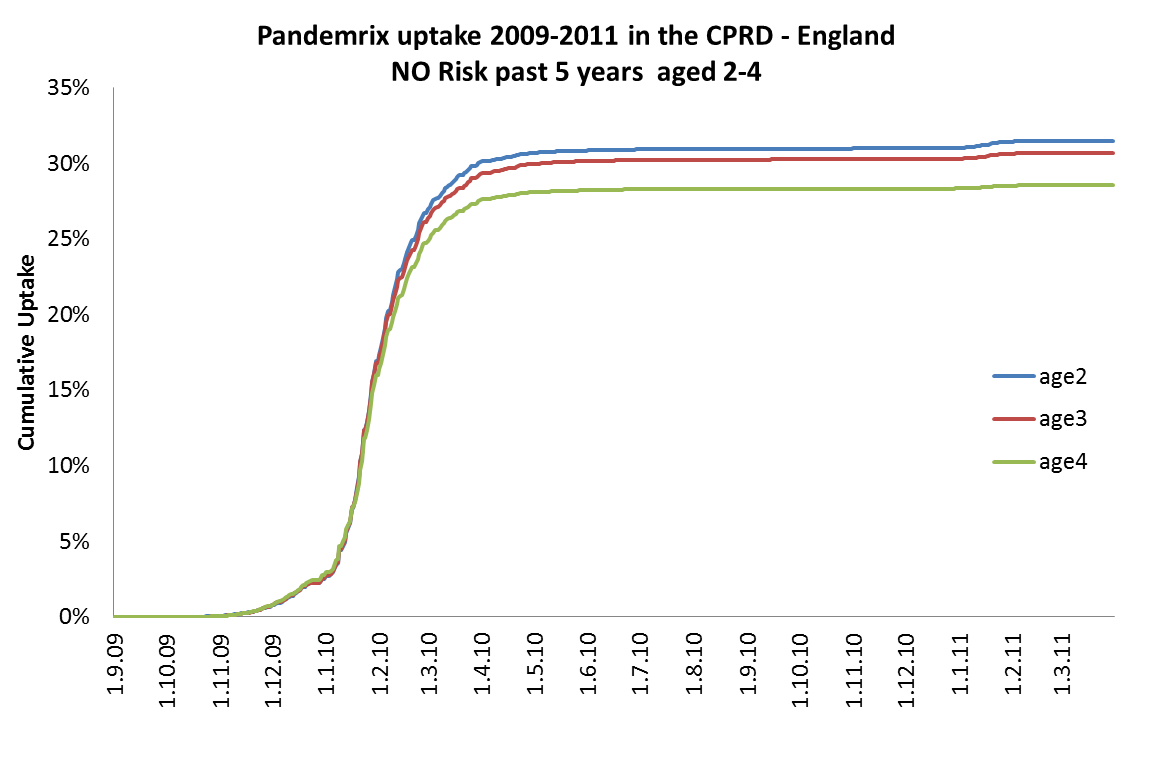

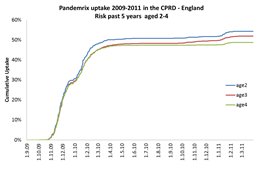


| 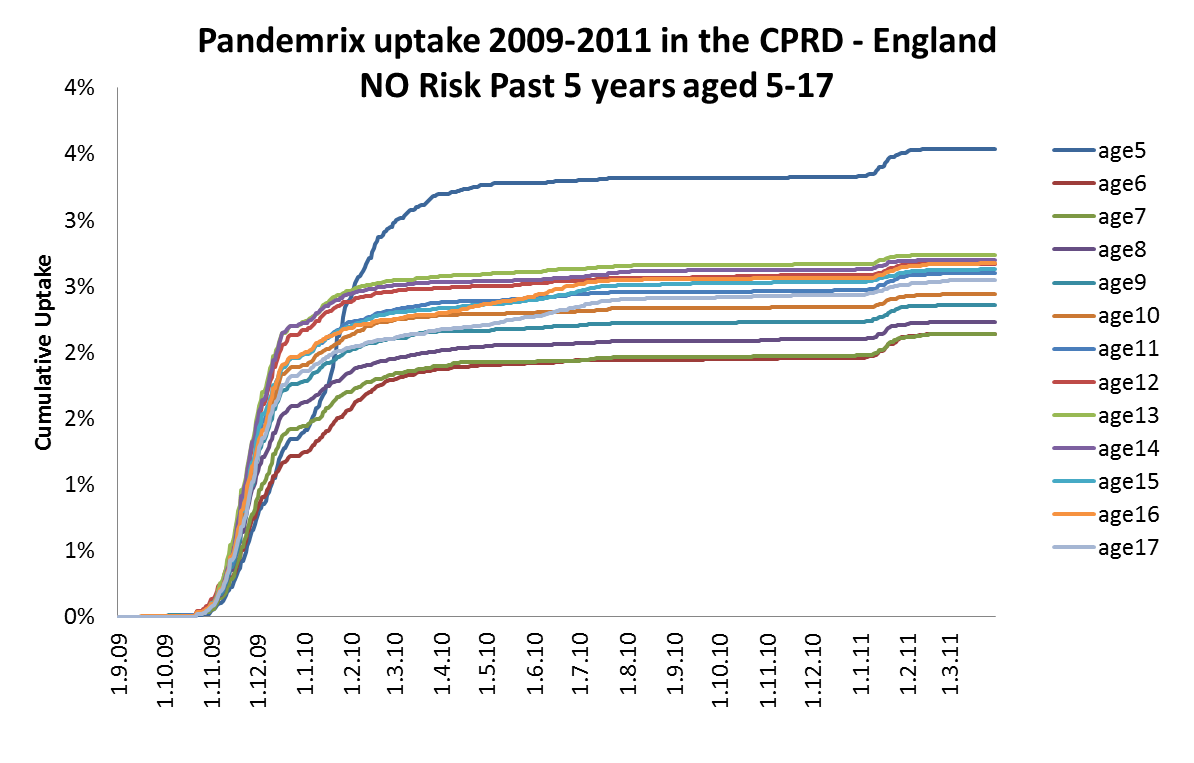 | 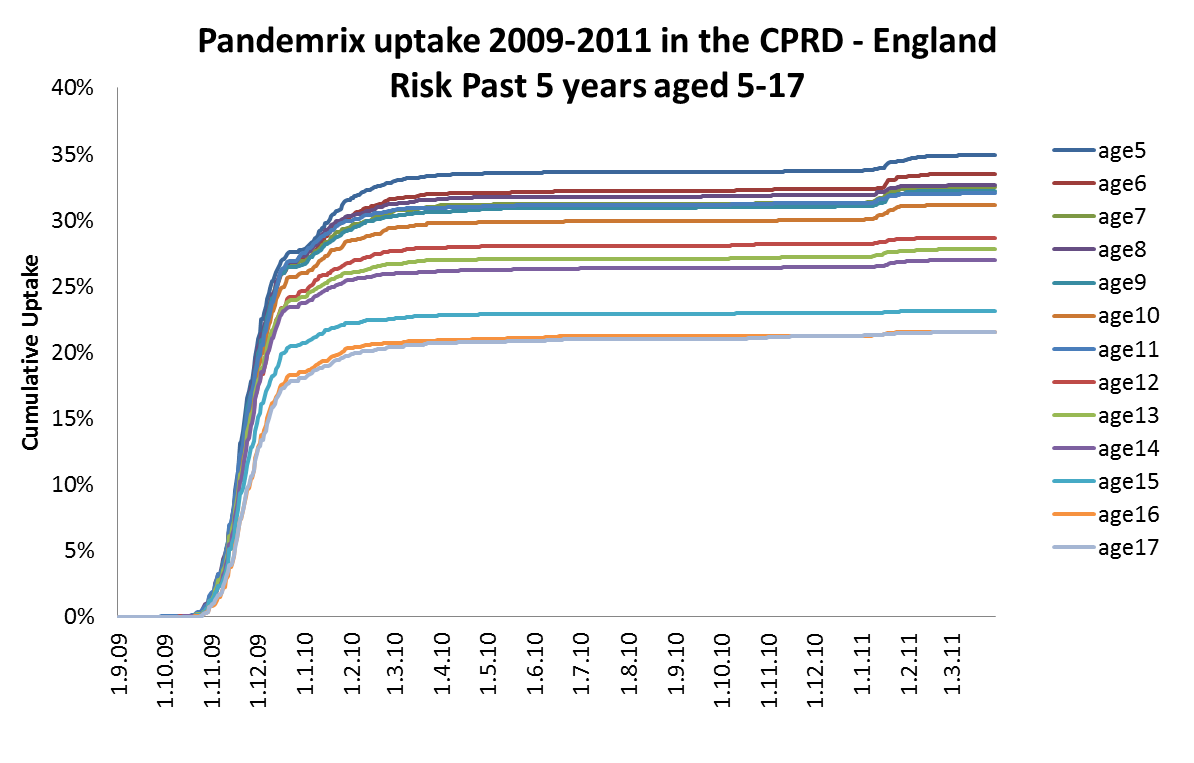 |
| --- | --- |

**2. Study aim, design and assessment of feasibility**

**2.1 Study aim**

The primary aim of the study is to estimate the attributable risk of narcolepsy post H1N1 influenza vaccine in children in England.

Secondary aims are as follows…

1. Test the hypothesis of an increased (or decreased risk), and estimate the risk, at any time post vaccination as well as for onset within <2 years, 2-3 years, 4-8 years.
2. Estimate the interval from onset to diagnosis and first health care contact to diagnosis in those vaccinated and unvaccinated.
3. Estimate the risk stratified by age at September 2009 <5, 5-16.

**2.2 Design**

The study design is the same as the previous HPA/PHE studies which is case-coverage (more details are given later). This design only requires ascertainment of case data and population level vaccine uptake data. The big potential confounders (i.e. related to vaccination and narcolepsy) are age and period. A possible confounder is having a co-morbidity for which vaccination is recommended as this is strongly related to likelihood of vaccine exposure and individuals with such risk factors may have be more/less likely to develop narcolepsy. All these confounders can be adjusted for in the case-coverage method. The case-coverage method is similar to a cohort design but the coverage data comes CPRD data. The study will be performed by identifying children with narcolepsy at 5 major sleep centres (details below) and independently obtaining vaccination history and further details of symptom onset, and whether the child is in a risk group for which fu vaccine is recommended from the GPs of these cases. Case and vaccine history ascertainment will be performed so as to minimise potential selection and recall bias. As only children’s sleep centres will be visited the study will not include some older children whom received the vaccine aged ≤19 but whom were diagnosed in adult centres.

Attributable fraction (AF) will be estimated as (RR-1)/RR, attributable cases as AF*Number of cases in the vaccine period and attributable risk as this number divided by the number of doses given to the population from which the cases arose. This population will be calculated using vaccine uptake data from the CPRD database and adjusted by the estimated proportion of narcolepsy cases captured (this will be approximated from HES data on case load at the 5 centres compared to the whole country).

**2.3 Sample size**

Vaccine uptake is about 23.6% in healthy children aged 6m to under 5 and of 33.7% to 42.3% in children in risk groups aged 2 to 19.

The original childhood study identified a 14 fold increased risk at any time following vaccination with 95% CI 4.3 to 48.5. Assuming the risk does not persist beyond about 2-3 years the odds ratio for any time since vaccination would be expected to be lower, but the attributable risk may be higher. If we assume no increased risk beyond about 3 years and a 10 fold increased risk prior to this then this will average at about a 4-5 fold risk over about 6-7 years follow up. Assuming the 5 study centres cover 67% of cases in the previous study and based on the number of cases seen in the previous study we would expect about 26×2/3 × 2 ≈ 35 cases (the ×2 relates to follow-up now 3 times as long as it was 2.5 years before, but with losing some older children from children’s sleep centres). The average coverage in the matched population will be about 12% (a little higher than the 8% in the previous study as more <5 year olds at vaccination will be included). Based on these numbers the precision for estimating ORs of 3,4,5,8 will be as shown below:

OR 95% CI

3 (1.4-6.3)

4 (2.0-8.0)

5 (2.5-9.8)

8 (4.1-15.5)

So there should be reasonable precision for the primary aim. Precision will be lower for sub-analyses (e.g. by age, time since vaccination).

**3. Definition of the population of interest**

**Location**: England

**Place of Narcolepsy diagnosis:** After analysing cases from HES in this period as an indication of the major centres and using centres from our childhood and adult narcolepsy; the following five sleep centres were identified.

These are as follows.

| **Centres** |  |  | | | |
| --- | --- | --- | --- | --- | --- |
| Evelina, St Thomas', London*  Oxford* |  |  | | | |
| Sheffield *  Papworth Hospital |  |  | | | |
| Leicester General Hospital  * **Xyrem Treatment centres**  As of 12 Dec 2016: 10 centres (which includes these 3 centres) can prescribe Xyrem (Xyrem is the last line in treatment for severe narcolepsy cases)   These have been identified by a NHS England Clinical commissioning group based on certain criterial: <https://www.england.nhs.uk/wp-content/uploads/2016/12/clin-comm-pol-16065P.pdf>  Xyrem can be prescribed outside of these centres for patients that have received the pandemic vaccine by applying to a DOH scheme <https://www.sleepsociety.org.uk/wp-content/uploads/2015/05/Ex-Gratia-Xyrem-Scheme-Summary-13-March-2015.pdf> | | | | |  |
|  | | |  |  | |

**Age:** between 4 and 19 and under at the time of diagnosis

In England much of the healthcare is spilt between paediatric (0-16 years) and adult care (16 years and above), although there is some overlap.

**Period for case ascertainment:** The full period is onset from April 1^st^ 2009 and diagnosed by a sleep centre by the time of the main visit.

The primary study period will be cases with onset of symptoms from April 1^st^ 2009 and with a date of diagnosis from this date to the date of the 1^st^ visit to that centre (key visit).

Note that “diagnosis” essentially means the case is identified at the centre visit as a case meeting the case definition.

**4. Case definition and timing of outcome**

Case selection will be carefully documented at each study site. The steps are as follows….

1. Screen databases and electronic clinic letters for all possible narcolepsy cases (HES – G47.4 and keywords *narco* *CSF*)
2. Remove cases clearly with onset outside the study period or that are not narcolepsy
3. Merge records, assign a unique ID and obtain scanned clinic letters and test results and summarise information in a database (appendix I).

The exact procedure in each centre will be documented.

***Expert review***

A panel of 4 experts will reviewed case notes and test results independently and classify cases using ICSD-2 criteria as…

1. definite narcolepsy with cataplexy,

2. definite narcolepsy without cataplexy,

3. probable narcolepsy

4. insufficient evidence to confirm diagnosis of narcolepsy.

5. not narcolepsy

Cases that clearly meet a case-definition of narcolepsy with ICSD-2 criteria (i.e. Excessive daytime sleepiness and positive MSLT) do not need to be reviewed in full. A selection of these cases will be shown to the experts to confirm that they are content that the diagnosis in such cases is definite.

Cases where 3 out of 4 experts agreed on the same classification will be assigned that classification. The final classification of the remaining cases will be agreed after discussing in a teleconference. All review will be done without knowledge of vaccination status.

The analysis will be based on all of categories 1-3 as power will be insufficient to stratify.

***Index dates***

Index dates to be recorded where possible will be

- **Date of 1^st^ symptoms** [We will also assign an earliest and latest date for sensitivity*].
- **Date of 1^st^ recorded consultation with health professional** [date of GP referral letter if available in case-note review, or when contacting GP first consultation with GP about sleep issues]
- **Date of referral to sleep centre**
- **Date 1^st^ consultation at Sleep Centre**
- **Date of Sleep test/ MSLT** [If done]
- **Date of diagnosis** [This will usually be the date of tests or if tests not done the date of a letter from the sleep centre with a diagnosis].

*The best estimate date will be used for the main analysis and the earliest and latest dates in sensitivity analyses. In general, we will use month mid-point (15^th^) unless a very specific date is given. For the uncertainty, we will base this on the comments with the follow general criteria: if the comment suggests onset was within the last year we will use +/- 1 month, within the last 2 years +/- 2months, 3 years +/- 3 months and >3 years +/- 6 months. If the best date we have was a health-care contact (GP) with no more information then this will be the latest date and the best date will be one month prior to this and earliest 2 months prior. We will use data from both the clinic notes and from the GP when they are contacted.

Primary index date is first symptoms. Sensitivity analyses will be first consultation with a health professional and date of diagnosis at the sleep centre. Date of first symptoms will be ascertained either from case notes or from GPs when they are written to ascertain vaccination history and risk group information.

**5. Exposure of interest and interval of exposure**

Pandemic H1N1 vaccine [Pandemrix] – given from October 2009 ascertained from the GP of the case. (appendix ). Note about 1% of the population received Celvepan – any such cases will be assessed separately. Any details on vaccination status recorded in the sleep centre notes / letters but not by the GP will not be used to assign vaccination status to avoid bias.

For time since vaccination we will use <2 year,2-3 years, and anytime (4 to about 8 years).

Coverage will be based on CPRD data previously extracted and shown in Figure 1, but updated to cover 6-11m and 1 year olds..

The primary risk period will be any time post vaccination.

**6. Key confounding variables**

For the case-coverage analysis confounding is addressed by matching coverage to cases based on the confounders. Clear confounders are age and calendar period and a possible confounder s risk group. These will be defined as follows:

Age (Age at September 2009 in years)

Calendar period (exact date)

Risk group for vaccination (yes/no).

The primary analysis will adjust for risk group in older children, but this will need to be risk group as at September 1^st^ 2009. A sensitivity analysis will de done not adjusting for risk group.

**7. Method of analysis**

**Case-coverage**: Here we compare the number of cases vaccinated with the expected number based on estimating the probability of vaccination. This can be done based on vaccine coverage. Vaccine coverage (within 2 years, 2-3 years and ever) will be matched by age in years (according to age in January 2010 to align with CPRD data), risk group and exact date of event. CPRD data are used to calculate vaccine uptake. To allow individual matching of coverage to cases the analysis will be performed using logistic regression with the outcome as vaccinated (1=yes 0 =no) in the cases and an offset for the log-odds of the matched coverage (ln(cover/(1-cover)). This is like the screening method of Farrington for estimating vaccine effectiveness. OR will approximate RR as the outcome is rare.

**8. Descriptive analysis**

This will be similar to that done for the previous childhood study.

- Flow diagram describing case finding by centre.
- Intervals between onset and presentation (line diagrams symptom---------GP--------Sleep C----MSLT----diagnosis).

BY vaccination status show…

- Cataplexy (yes/no)
- Gender (male/female)
- Co-morbidities (yes/no)
- Age at September 2009 (<5,5-11,12-18)
- Age at Onset (<5,5-11,12-18)
- Age at Diagnosis (<5,5-11,12-18)
- Calendar period of various event dates (Year/Month)
- HLA type
- CSF measurement
- Diagnostic Category – 1,2,3

Comparison of delays to diagnosis between vaccinated and unvaccinated will be done by survival analysis and only including cases with onset from April 2009-March 2012 whom are diagnosed within 5 years.

**9. Sensitivity analysis / stratification**

The only stratification will be by age at September 2009 (<5, >=5)

As with the previous analysis of the childhood cases a core model will be defined and sensitivity analyses performed around this model.

The core model will be as follows:

- Period of cases: Onset October 2009 to diagnosed at the sleep centre by the centre visit date
- Index date: date of first symptoms
- Exposure period: Ever
- Adjustment: matching by age (years), period (exact date) and risk group

Sensitivity around the core model will be:

- Index date: first health care contact, diagnosis
- Exposure: < 2 years, <4 years, 2-3 years,4-8 years
- Adjustment: no risk group adjustment
- Vaccine coverage: increase / decrease by a relative 20% (e.g. 10% goes to 12% or 8%)
- Assuming all individuals are in risk groups
- Using earliest and latest onset dates based on uncertainty if this leads to change in vaccination status of any cases
- Just including cases registered at the GP practice in September 2009

**10. Limitations and dealing with them**

Numbers may be too small for stratified analyses. This is an issue of interpretation. The sensitivity analyses aim to address some limitations such as inaccuracies in uptake data and uncertainty on onset and risk group status. Another limitation may be missing data on vaccination history given the time since exposure. Children may have moved GP but previous GP details will be traced using the PDS system and contacted when necessary. The Child Health Information System (CHIS) also contains some vaccine information for children under 16 and these systems will be contacted if necessary. If this is not missing it could introduce bias. It will therefore be important to find out when children registered with the practice.

**11. Data management**

Data from case note review and GP questionnaires will entered into an Access database and exported into Excel. Analysis will be in Stata version 12.

**12. Significance level**

5% significance will be used and 95% confidence intervals calculated.

**13. Data Security**

- The study will comply with the six Caldicott principles governing the use of person-identifiable information. PHE has had systems in place for several years to show compliance with Caldicott principles. The immunisation department has a Caldicott monitor which carries out monthly audits and the site has an overall Caldicott audit, all of which are monitored by the Health Care Commission.
- The information collected will be put into a password protected Access database which will be located in a restricted access folder on the PHE network. The PHE network is protected by a secure firewall to prevent outside access to the organisations network.
- Once information has been collected for GPs Hospital NHS number/names will be removed and the study number used.
- The form the GP sends back will only have study number present
- Identifiers (NHS number/case note number) taken to hospitals to carry out case note review will be held in a password protected excel file on the encrypted laptop
- Transfers of PATIENT data outside the secure area will be encrypted in line with Cabinet Office requirements.
- PHE laptops used to access the data employ full disk encryption using McAfee Endpoint Encryption, managed by PHE IT. See: PHE SQL Database Servers (including Porton SQL Clusters) & PID_Fileshare_SLSP
- Electronic media will be erased using a software package. If the equipment is to be discarded, storage media such as fixed disks will be removed from computers and rendered unreadable by destruction.
- The study is under the National Information Governance Board for Health and Social Care (NIGB) (PIAG ref: PIAG 03-(c)/2001) which allows access to patient identifiable information for vaccine safety monitoring purposes.
- PHE does have stringent data security measures in place and are registered under the Data Protection Act (registration number: Z7749250) and

See appendix 2 for the data transfer flow diagram.

**14. Changes to the analysis plan (after data received)**

Changes will be documented in this document and dated.

Change Date

**References**

1. Longstreth WT Jr, Koepsell TD, Ton TG, et al. The epidemiology of narcolepsy. Sleep. 2007 Jan 1;30(1):13–26. Review.
2. Partinen M, Saarenpää-Heikkilä O, Ilveskoski I, et al.Increased incidence and clinical picture of childhood narcolepsy following the 2009 H1N1 pandemic vaccination campaign in Finland. PLoS One. 2012;7(3):e33723. Epub 2012 Mar 28. PubMed PMID: 22470463;PubMed Central PMCID: PMC3314680.
3. Nohynek H, Jokinen J, Partinen M, et al. AS03 adjuvanted AH1N1 vaccine associated with an abrupt increase in the incidence of childhood narcolepsy in Finland. PloS One 2012; 7(3):e33536.
4. Jokinen J, Nohynekm H, Honkanenm J, et al. Working paper: Association between the pandemic vaccine and narcolepsy in adults. 2013 http://urn.fi/URN:ISBN:978-952-245-921-3. Accessed January 22, 2016.
5. Miller E, Andrews N, Stellitano L, et al. Risk of narcolepsy in children and young people receiving AS03 adjuvanted pandemic A/H1N1 2009 influenza vaccine: retrospective analysis. BMJ 2013;346:f794.
6. Stowe J, Andrews N, Kosky C, Dennis G, Eriksson S, Hall A, Leschziner G, Reading P, Shneerson JM, Donegan K, Miller E. (2016). Risk of Narcolepsy after AS03 Adjuvanted Pandemic A/H1N1 2009 Influenza Vaccine in Adults: A Case-Coverage Study in England. Sleep. 2016 May 1;39(5):1051-7.
7. Persson I, Granath F, Askling J, et al. Risks of neurological and immune-related diseases, including narcolepsy, after vaccination with Pandemrix: a population- and registry-based cohort study with over 2 years of follow-up. J Intern Med. 2014 Feb;275(2):172-90
8. Szakács A, Darin N, Hallböök T. Increased childhood incidence of narcolepsy in western Sweden after H1N1 influenza vaccination. Neurology. 2013 Apr 2;80(14):1315-21
9. Dauvilliers Y, Arnulf I, Lecendreux M, et al. Increased risk of narcolepsy in children and adults after pandemic H1N1 vaccination in France. Brain 2013;136(Pt 8):2486–96.
10. O'Flanagan D, Barret AS, Foley M, et al. Investigation of an association between onset of narcolepsy and vaccination with pandemic influenza
11. vaccine, Ireland April 2009-December 2010. Euro Surveill 2014 May 1;19(17):15–25.
12. Heier MS, Gautvik KM, Wannag E, et al. Incidence of narcolepsy in Norwegian children and adolescents after vaccination against H1N1 influenza A. Sleep Med 2013;14(9): 867–71.

Appendix 1: Data collection sheet


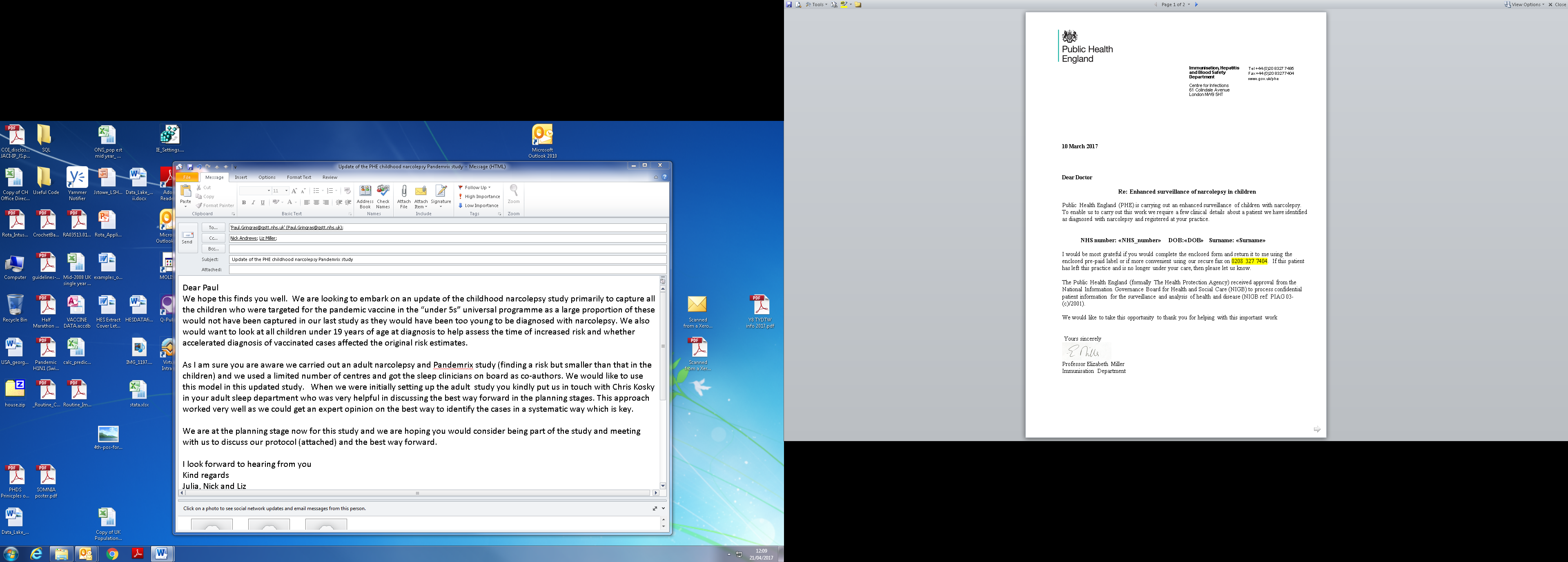


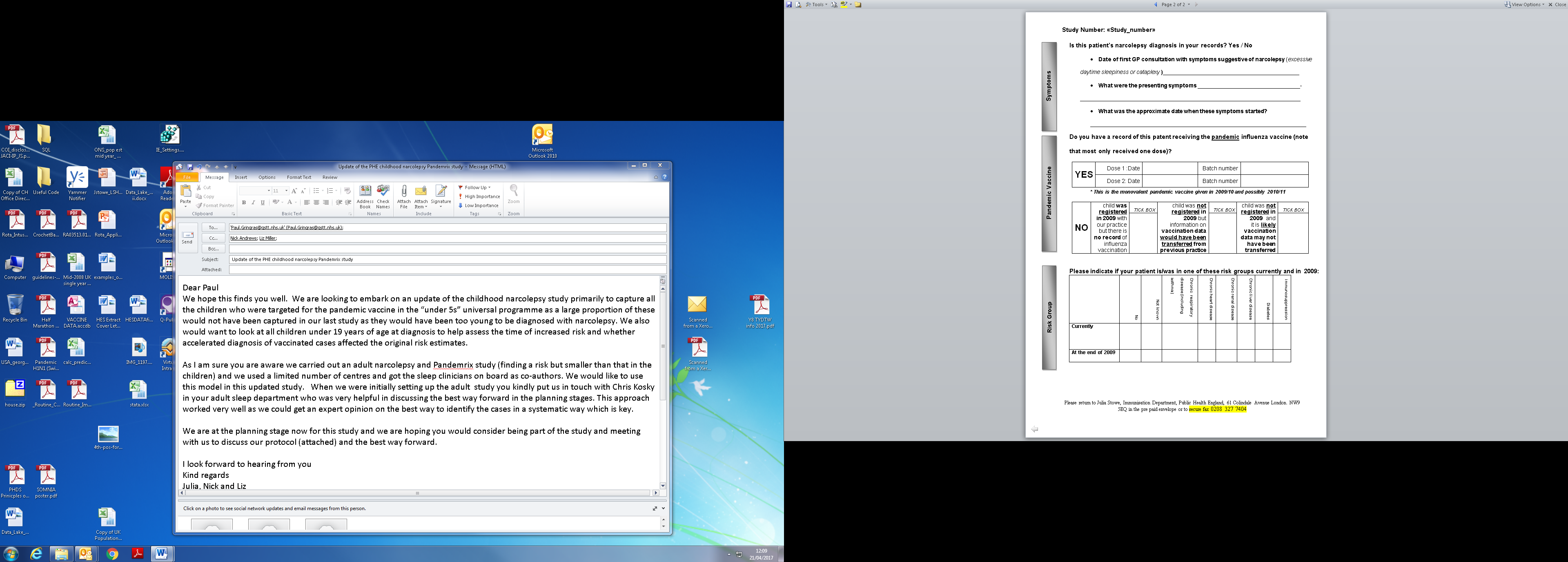


Appendix 2:


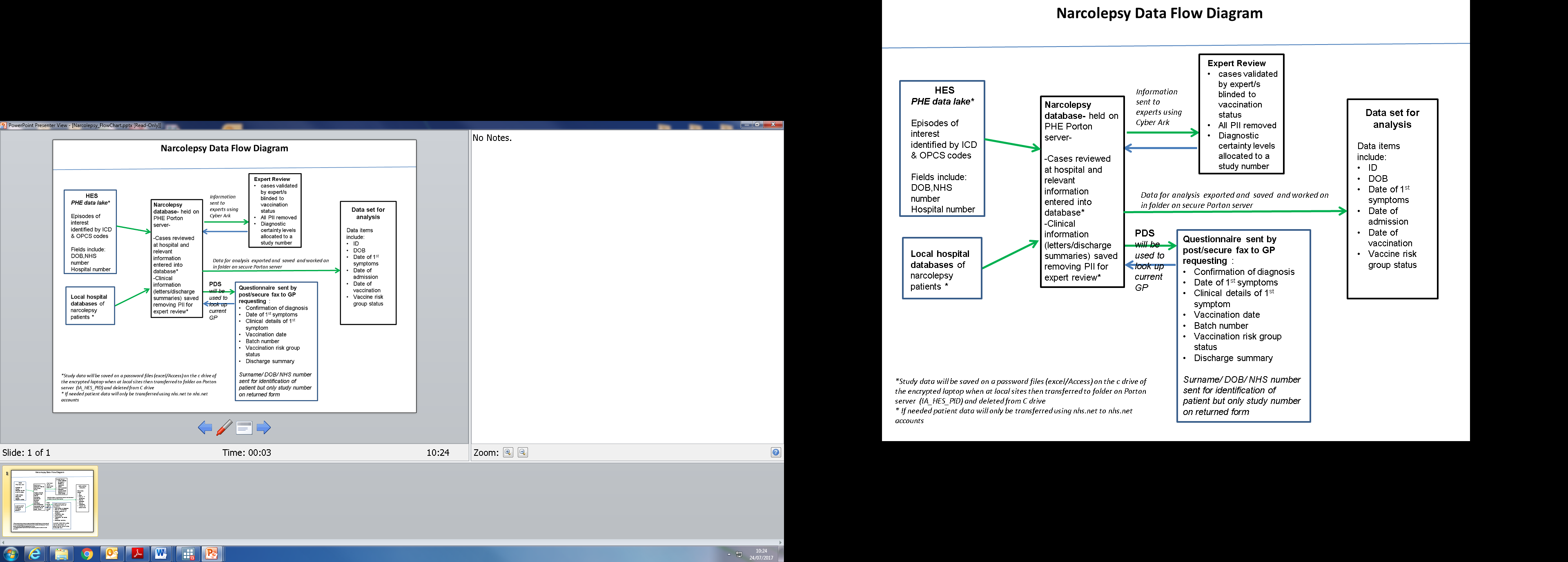

Supplement: S1 Text — (DOC) [file pmed.1003225.s002.doc]
