# Supplementary figures and images for: Reassessment of the risk of narcolepsy in children in England 8 years after receipt of the AS03-adjuvanted H1N1 pandemic vaccine: A case-coverage study
Source: PLoS Med. 2020 Sep 14;17(9):e1003225. doi: 10.1371/journal.pmed.1003225 (PMC7489954; doi:10.1371/journal.pmed.1003225)

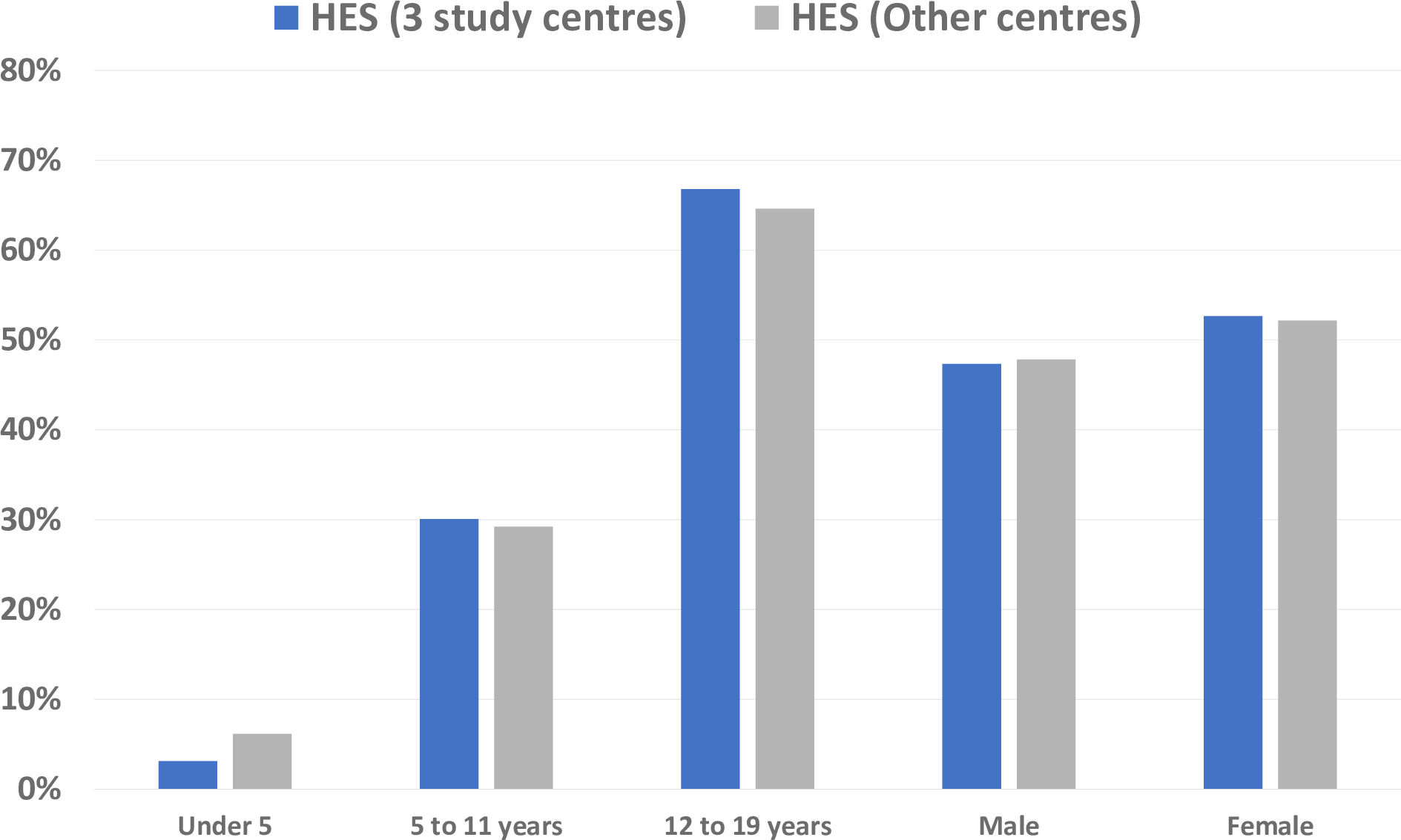

Supplement: S1 Fig — HES, hospital episode statistics; ICSD-3, International Classification of Sleep Disorders Third Edition. (TIF) [file pmed.1003225.s003.tif]
