# Supplementary material for: Reassessment of the risk of narcolepsy in children in England 8 years after receipt of the AS03-adjuvanted H1N1 pandemic vaccine: A case-coverage study
Source: PLoS Med. 2020 Sep 14;17(9):e1003225. doi: 10.1371/journal.pmed.1003225 (PMC7489954; doi:10.1371/journal.pmed.1003225)
Supplement: S2 Fig — (DOCX) [file pmed.1003225.s004.docx]

**Coverage data**

Coverage data for Pandemrix was obtained from the Clinical Practice Research Datalink (CPRD) by age (at January 2010) and risk group status. As expected coverage is highest in those targeted with the vaccine (aged <5 and in risk groups). Most vaccination was in 2009-10 with only small increases in 2010-11 when Pandemrix was offered during a temporary shortage of seasonal influenza vaccine. These coverage data were used to obtain the matched coverage for the cases based on age, risk group status and index date.

To obtain estimates of the probability of vaccination in the various time intervals up to 8 years prior to an index date the cumulative coverage was matched at the date at each end of the time interval (e.g. 1 year before the index date and 2 years before the index date) and the difference in coverage calculated.

**
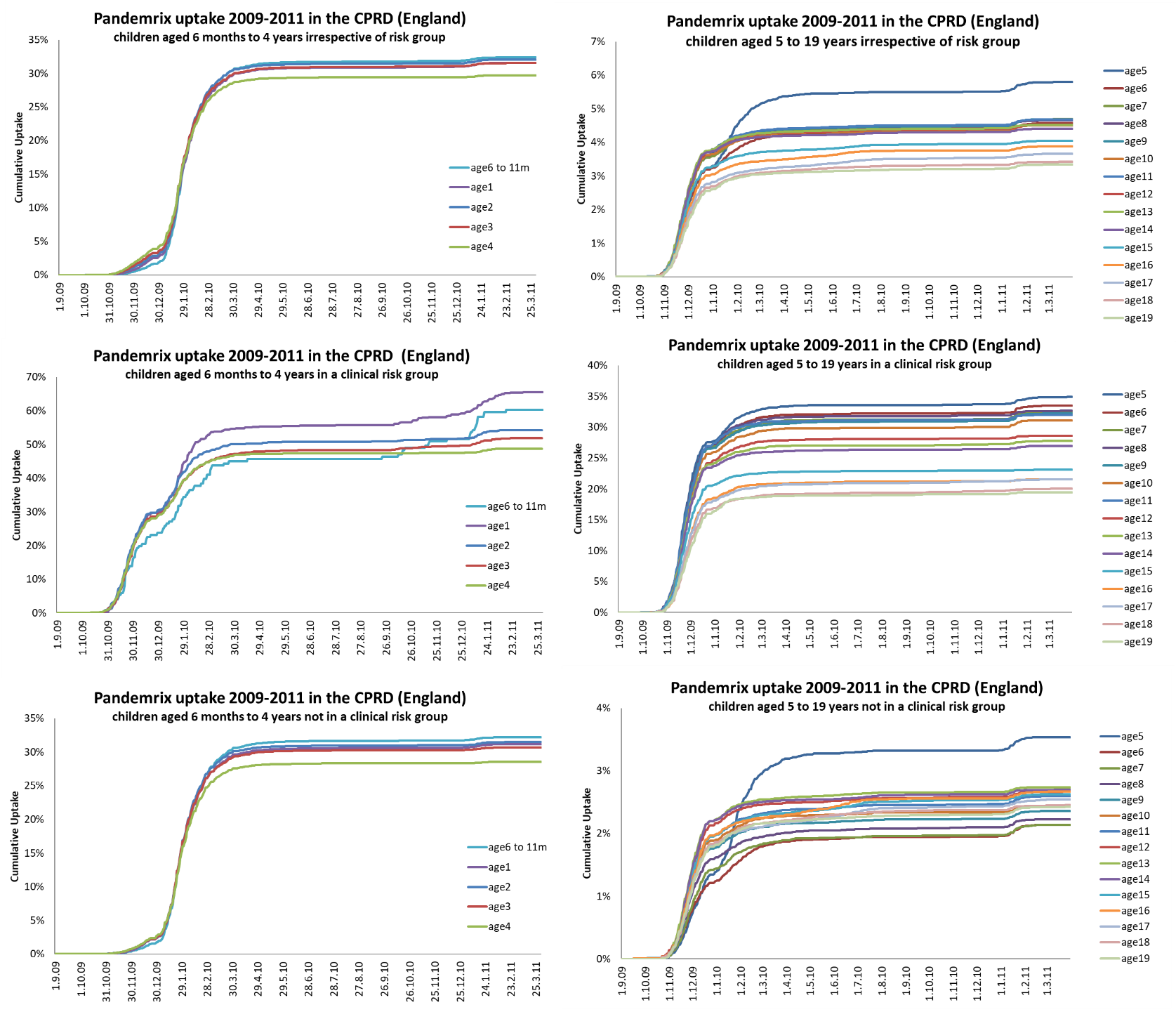
**
