# Supplementary material for: Reassessment of the risk of narcolepsy in children in England 8 years after receipt of the AS03-adjuvanted H1N1 pandemic vaccine: A case-coverage study
Source: PLoS Med. 2020 Sep 14;17(9):e1003225. doi: 10.1371/journal.pmed.1003225 (PMC7489954; doi:10.1371/journal.pmed.1003225)
Supplement: S1 Data — (DOCX) [file pmed.1003225.s005.docx]

**Estimation of doses given to the population covered by the study centres**

| Age | ONS Population 2009  [A] | Coverage (CPRD)  [B] | Estimated doses given to England population  [C=A × B] | Estimated doses to study population (41%)  [D = 0.41 ×C] |
| --- | --- | --- | --- | --- |
| 6-11m | 331403 | 32.4% | 107298 | 43992 |
| 1 | 667271 | 31.6% | 210697 | 86386 |
| 2 | 643986 | 32.1% | 206477 | 84656 |
| 3 | 630621 | 31.5% | 198847 | 81527 |
| 4 | 607221 | 29.7% | 180449 | 73984 |
| 5 | 597452 | 5.8% | 34621 | 14194 |
| 6 | 578513 | 4.6% | 26465 | 10851 |
| 7 | 566002 | 4.7% | 26541 | 10882 |
| 8 | 577340 | 4.7% | 26860 | 11013 |
| 9 | 592465 | 4.7% | 27770 | 11386 |
| 10 | 610274 | 4.5% | 27560 | 11299 |
| 11 | 618336 | 4.7% | 28975 | 11880 |
| 12 | 635554 | 4.5% | 28647 | 11745 |
| 13 | 633426 | 4.5% | 28631 | 11739 |
| 14 | 631249 | 4.4% | 27795 | 11396 |
| 15 | 651381 | 4.0% | 26377 | 10815 |
| 16 | 653819 | 3.9% | 25383 | 10407 |
| 17 | 673880 | 3.7% | 24705 | 10129 |
| 18 | 693106 | 3.4% | 23709 | 9721 |
| 19 | 682829 | 3.3% | 22802 | 9349 |
| Total 6m-18* | 11593299 | 11.1% | 1287807 | 528001 |

*for the total doses 19 year olds were not included because it is unlikely those vaccinated aged 19 would be diagnosed whilst still aged 19.
