# Supplementary material for: Reassessment of the risk of narcolepsy in children in England 8 years after receipt of the AS03-adjuvanted H1N1 pandemic vaccine: A case-coverage study
Source: PLoS Med. 2020 Sep 14;17(9):e1003225. doi: 10.1371/journal.pmed.1003225 (PMC7489954; doi:10.1371/journal.pmed.1003225)
Supplement: Supplementary file 5 [file pmed.1003225.s001.docx]

Re-assessment of the risk of narcolepsy in children in England eight years after receipt of the ASO3-adjuvanted H1N1 pandemic vaccine: a case-coverage analysis

	Item No	Recommendation	Section in manuscript	Paragraph
Title and abstract	1	(*a*) Indicate the study's design with a commonly used term in the title or the abstract	Title	
		(*b*) Provide in the abstract an informative and balanced summary of what was done and what was found	Abstract	
**Introduction**				
Background/rationale	2	Explain the scientific background and rationale for the investigation being reported	Introduction	1-3
Objectives	3	State specific objectives, including any prespecified hypotheses	Introduction	4
**Methods**				
Study design	4	Present key elements of study design early in the paper	Methods	Protocol and preplanned analysis provided in Supporting information S1
Setting	5	Describe the setting, locations, and relevant dates, including periods of recruitment, exposure, follow-up, and data collection	Methods	Case ascertainment and validation
		Case-coveragestudy —Give the eligibility criteria, and the sources and methods of case ascertainment and control selection. Give the rationale for the choice of cases and controls	Methods	Cases selected as per case control study. Described under case ascertainment and validation
		Case-coverage study —For matched studies, give matching criteria and the number of controls per case	Methods	Statistical Analysis section. Coverage population matched on age and risk factors (in case coverage method population can include cases) Numbers given in S5 Supplementary information
Variables	7	Clearly define all outcomes, exposures, predictors, potential confounders, and effect modifiers. Give diagnostic criteria, if applicable	Methods	Case definition and validation provided in case asertainment section of methods
Data sources/ measurement	8*	For each variable of interest, give sources of data and details of methods of assessment (measurement). Describe comparability of assessment methods if there is more than one group	Methods	Method for asigning key index dates (symptom onset, health care contact and diagnosis) described in Index dates section of methods
Bias	9	Describe any efforts to address potential sources of bias	Methods	Matching and use of different index dates
Study size	10	Explain how the study size was arrived at	Methods	Described in protocol and pre planned analysis S1
Quantitative variables	11	Explain how quantitative variables were handled in the analyses. If applicable, describe which groupings were chosen and why	Methods	Age grouping in analysis (<5 years and 5-19 years) reflected study aim to compare risk in young and older children
Statistical methods	12	(*a*) Describe all statistical methods, including those used to control for confounding	Methods	Statistical analysis section of methods
		(*b*) Describe any methods used to examine subgroups and interactions	Methods	Age stratification as above
		(*c*) Explain how missing data were addressed	Methods	Cases with missing vaccination hstory (n=2) excluded
		Case-coverage study —If applicable, explain how matching of cases and controls was addressed	Methods	Matched for age and risk group status
		(*e*) Describe any sensitivity analyses	Methods	Described in statistical analysis section of methods
**Results**				
Participants	13*	(a) Report numbers of individuals at each stage of study—eg numbers potentially eligible, examined for eligibility, confirmed eligible, included in the study, completing follow-up, and analysed	Results	Flow diagram of numbers in Figure 1 [illegible]
